# Three-year outcomes of surgical bleb revision with mitomycin C for early scarring bleb after trabeculectomy

**DOI:** 10.1007/s10384-024-01142-7

**Published:** 2024-11-21

**Authors:** Risa Caraher-Masuda, Mari Sakamoto, Mina Okuda, Fumio Takano, Sotaro Mori, Kaori Ueda, Akiyasu Kanamori, Yuko Yamada-Nakanishi, Makoto Nakamura

**Affiliations:** 1 Division of Ophthalmology, Department of Surgery, Kobe University Graduate School of Medicine, 7-5-2, Kusunoki-cho, Chuo-ku, Kobe, 650-0017 Japan; 2 Kanamori Eye Clinic, Kobe, Japan

**Keywords:** Bleb revision, Trabeculectomy, Needling, Glaucoma

## Abstract

**Purpose:**

To report the 3-year outcomes of surgical bleb revision (SBR) with mitomycin C (MMC) for early scarring bleb after trabeculectomy (TLE).

**Study design:**

Retrospective observational study.

**Methods:**

We included glaucoma patients aged ≧ 18 who underwent SBR with MMC within 6 months of their first TLE at Kobe University Hospital and were followed for at least 6 months. The primary outcome measure was the three-year success rate of SBR. We defined surgical success as: intraocular pressure (IOP) reduction ≧ 20% from baseline and 5 ≦ IOP ≦ 18 mmHg. Failure was defined when the IOP deviated from the criteria, when the eye required additional glaucoma surgery, and when the eye lost light perception. Complete success (CS) was success without glaucoma medications and qualified success (QS) was success with glaucoma medications. The secondary outcome measures included IOP, the number of glaucoma medications, mean deviation (MD), best corrected visual acuity (BCVA), corneal endothelial cell density (ECD), and surgical complications.

**Results:**

Sixty-eight eyes of 68 patients were analyzed. The median interval between initial TLE and SBR was 2 months. Overall success rate at three-year after SBR were 45.1% and 9.6% for QS and CS, respectively. A greater number of medications used before TLE was a contributing factor to failure (*P* = 0.02). 22 eyes (32.4%) underwent additional glaucoma surgery, and 41 eyes (60.3%) were spared from additional glaucoma surgery within 3 years after SBR. The median IOP decreased form 24.0 mmHg to 11.0 mmHg 3 years after SBR, and the number of medications decreased from 4 to 2 (*P* < 0.01). MD remained unchanged, but BCVA and ECD decreased at 3years postoperatively. There were no serious complications of SBR.

**Conclusion:**

SBR may be an effective treatment option for early scarring blebs after TLE but is unsuccessful in eyes that have used many glaucoma medications prior to TLE.

**Supplementary Information:**

The online version contains supplementary material available at 10.1007/s10384-024-01142-7.

## Introduction

Trabeculectomy (TLE) is the gold standard for glaucoma surgery because of its excellence in lowering intraocular pressure (IOP). The main cause of TLE failure is postoperative scarring of the bleb, hence long-term maintenance of a functional bleb is the key to TLE success. Although antimetabolites such as mitomycin C (MMC) are used to prevent bleb scarring, scarring already begins in the early postoperative period. In a previous report using polarized optical coherence tomography (OCT) to scrutinize bleb morphology in the early postoperative period after TLE, fibrosis of the internal tissues was already present at 2 weeks postoperatively, prior to the deterioration of bleb function [1]. Other studies using anterior segment OCT report that high reflectivity of the bleb wall at 2 weeks post-TLE, which may reflect fibrosis of the bleb wall, was a risk factor for bleb failure [2, 3]. Therefore, management of bleb scarring that develops early after TLE is essential for long-term maintenance of bleb function.

There is a variety of methods for managing early postoperative bleb scarring, including the use of topical or oral steroid medications, ocular massage, bleb needling and surgical bleb revision (SBR), with methods varying from surgeon to surgeon and institution to institution. Bleb needling may be performed more often than procedures in the operating room as it can be performed easily on a slitlamp and can be repeated. Bleb needling does not require a conjunctival incision and sutures, and is less invasive to the conjunctiva than surgical procedures. However, the success rate of needling varies widely depending on the technique and definition of success, it is reported to be approximately 20–80% [4–15]. In addition, bleb needling is a partially blind technique that can cause unexpected bleeding and may contribute to further scarring of the bleb [16]. Until about 10 years ago, we used to do bleb needling for early scarring blebs in Kobe University. However, the results of needling were poor, and over time, surgeons began to make a small incision to the conjunctiva far behind the flap, apply MMC, and insert a blunt needle under the flap into the anterior chamber while detaching the adhesions to resume aqueous humor outflow, SBR. In the early days, bleb needling was performed first, and whenever it wasn’t effective, SBR was performed. Gradually, however, the surgeons felt that SBR was more reliable than bleb needling in revising the scarred bleb, and began to perform SBR alone. Currently, at Kobe University, in almost all cases of early postoperative bleb scarring we perform SBR in the operating room. Although there are many reports on SBR, most of them are on SBR for bleb failures after over a year following the initial TLE, as well as persistent hypotony, late bleb leaks, and dysesthesia after TLE [17–24]. To our knowledge, there are few reports on SBR for early postoperative bleb scarring. We report the results of our SBR performed at very early postoperative time after TLE.

## Methods

This was a retrospective observational study. The study protocol was approved by the Institutional Review Board of Kobe University Hospital (approval no. B220131) and was conducted following the tenets of the Declaration of Helsinki.

We reviewed the medical charts of consecutive patients with glaucoma who underwent SBR with MMC for early scarring bleb after initial TLE at Kobe University Hospital between January 2015 and December 2019.

We included (1) patients aged ≧18, (2) those who underwent SBR within 6 months of initial TLE because of elevation of IOP due to bleb scarring, and (3) those with a minimum follow-up period of six months after SBR. One eye per patient was included. If both eyes met the inclusion criteria, the first operated eye was included. We excluded (1) SBR for leaking blebs, (2) SBR for infected blebs, and (3) SBR augmented with amniotic membrane. The following data were collected from the medical records: age, gender, type of glaucoma, history of prior surgery in the included eye, complications of the TLE and SBR, best corrected visual acuity (BCVA), mean deviation (MD) for Humphrey visual field, and corneal endothelial cell density (ECD) before TLE, SBR and at the last follow-up, IOP measured by Goldmann applanation tonometry and number of glaucoma medications used before TLE, before SBR, at post-SBR month 1, 3, 6, 12, 24, and 36, and additional glaucoma surgery after SBR. The use of combination eye drops, or oral acetazolamide was counted as two medications. The primary outcome measure was the three-year success rate of SBR analyzed by the Kaplan-Meier method. We defined surgical success as: IOP reduction ≧ 20% from pre-TLE IOP and 5 ≦ IOP ≦ 18 mmHg. Failure was defined when the IOP deviated from the above criteria on two consecutive post-operative visits, when the eye required additional glaucoma surgery to control IOP, and when the eye lost light perception. Complete success (CS) was defined when the IOP criteria were met without any glaucoma medications; qualified success (QS) was with glaucoma medications. The secondary outcome measures included postoperative IOP and the number of glaucoma medications needed, post operative BCVA, postoperative MD, postoperative ECD, and surgical complications. The Wilcoxon matched-pairs signed-rank test was used to compare pre and post operative data. Postoperative changes in IOP and the number of glaucoma medications were analyzed using a mixed-effect model. All statistical analyses were performed using MedCalc Statistical Software version 20.010 (MedCalc Software Ltd) and EZR [25], a modified version of R commander, with type I error for significance set at *P* < 0.05.

## Surgical technique (Fig. 1)

In all cases, initial TLE was performed with fornix-based incision at superior conjunctiva with application of 0.04% MMC for 3 minutes. SBR was conducted in the operating room using a surgical microscope. After administering topical anesthesia (2% lidocaine) to the sub-Tenon space, a clear cornea stay suture (6-0 vicryl) was placed to rotate the eye downwards to expose the superior conjunctiva. 2% lidocaine was injected under the conjunctiva posteriorly away from the bleb to check the surrounding adhesions. Then a small incision up to 3 mm wide was made in the conjunctiva posterior and distal to the existing TLE flap to expose the Tenon and sclera. The Tenon and sclera posterior to the incision site were bluntly dissected toward the fornix, and sponges soaked in 0.04% MMC were applied to the posterior sub-Tenon space for 3 minutes. After washing with balanced salt solution, incision and dissection of the scarred conjunctiva was conducted anteriorly to identify the original TLE flap. Scarred tissue was excised selectively. The existing sutures left on the TLE flap were removed. A blunt needle was inserted under the flap and advanced into the anterior chamber until aqueous humor outflow was observed. The scleral flap was left open without sutures except in cases of excessive aqueous outflow and poor anterior chamber formation. Intraoperative bleeding was stopped with bipolar diathermy as necessary. Finally, the conjunctiva was continuously sutured with 10-0 nylon. Postoperative topical therapy consisted of betamethasone and antibiotic eye drops four to six times a day for one to three months, depending on the bleb status and IOP, then tapered thereafter.

**Fig. 1:**
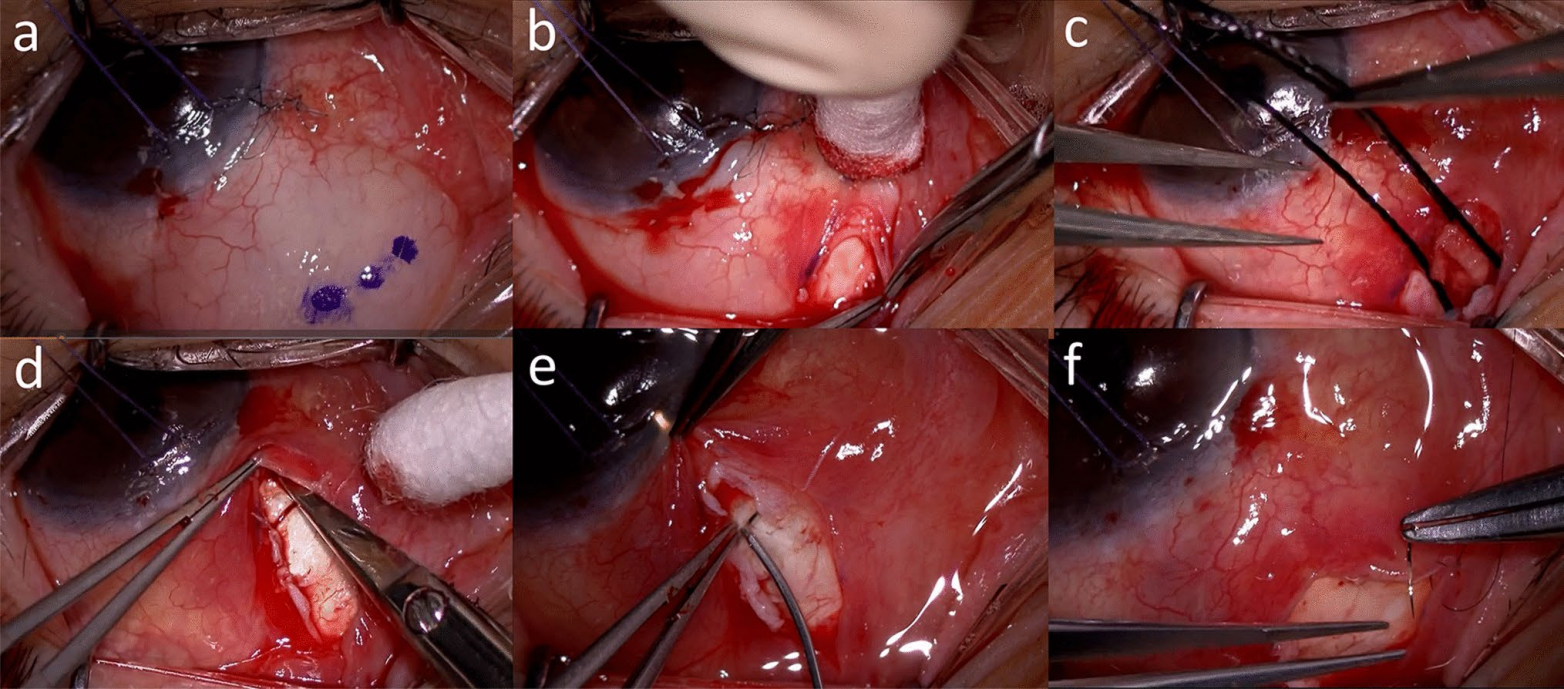
**a** After marking the conjunctiva for the incision, 2% lidocaine was injected under conjunctiva posteriorly away from the bleb. **b** The Tenon and sclera posterior to the incision site were bluntly dissected toward the fornix. **c** Sponges soaked in 0.04% mitomycin C (MMC) were applied to the posterior sub-Tenon space for 3 minutes. **d** Incision and dissection of the scarred conjunctiva was proceeded anteriorly to identify the original scleral flap. **e** A blunt needle was inserted under the flap and advanced into the anterior chamber until aqueous humor outflow was observed. **f** Finally, the conjunctiva was continuously sutured with 10-0 nylon

## Results

A total of 68 eyes of 68 patients with glaucoma were included. The characteristics of the patients are shown in Table 1. The median (interquartile range, IQR) interval from initial TLE to SBR was 2 months (2.0–3.0).

**Table 1:** Patient characteristics

Total number of eyes/patients	n = 68
Age (years old)	70.0 (61.0–77.0)^a^
Sex (male, %)	41 (60.3)
Intervlas from TLE to SBR (months)	2.0 (2.0–3.0)^a^
Types of glaucoma (n, %)	
POAG	31 (45.6)
Secondary	33 (48.5)
Exfoliative Glaucoma	19 (27.9)
Uveitis	11 (16.2)
Neovascular Glaucoma	2 (2.9)
Steroid	1 (1.5)
Congenital	3 (4.4)
PACG	1 (1.5)
Past Operations (n, %)	
PEA+IOL	18 (26.7)
TLO	9 (13.2)
TLO+PEA+IOL	9 (13.2)
PPV+PEA+IOL	4 (5.8)
Trabectome	3 (4.4)
GSL+PEA+IOL	1 (1.5)

*TLE* trabeculectomy, *SBR* surgical bleb revision, *POAG* primary open angle glaucoma, *PACG* primary angle closure glaucoma, *PEA* phacoemulsification and aspiration, *IOL* intraocular lens, *TLO* trabeculotomy, *PPV* pars plana vitrectomy, *GSL* goniosynechialysis

^a^Data are expressed as median (interquartile range)

**Fig. 2:**
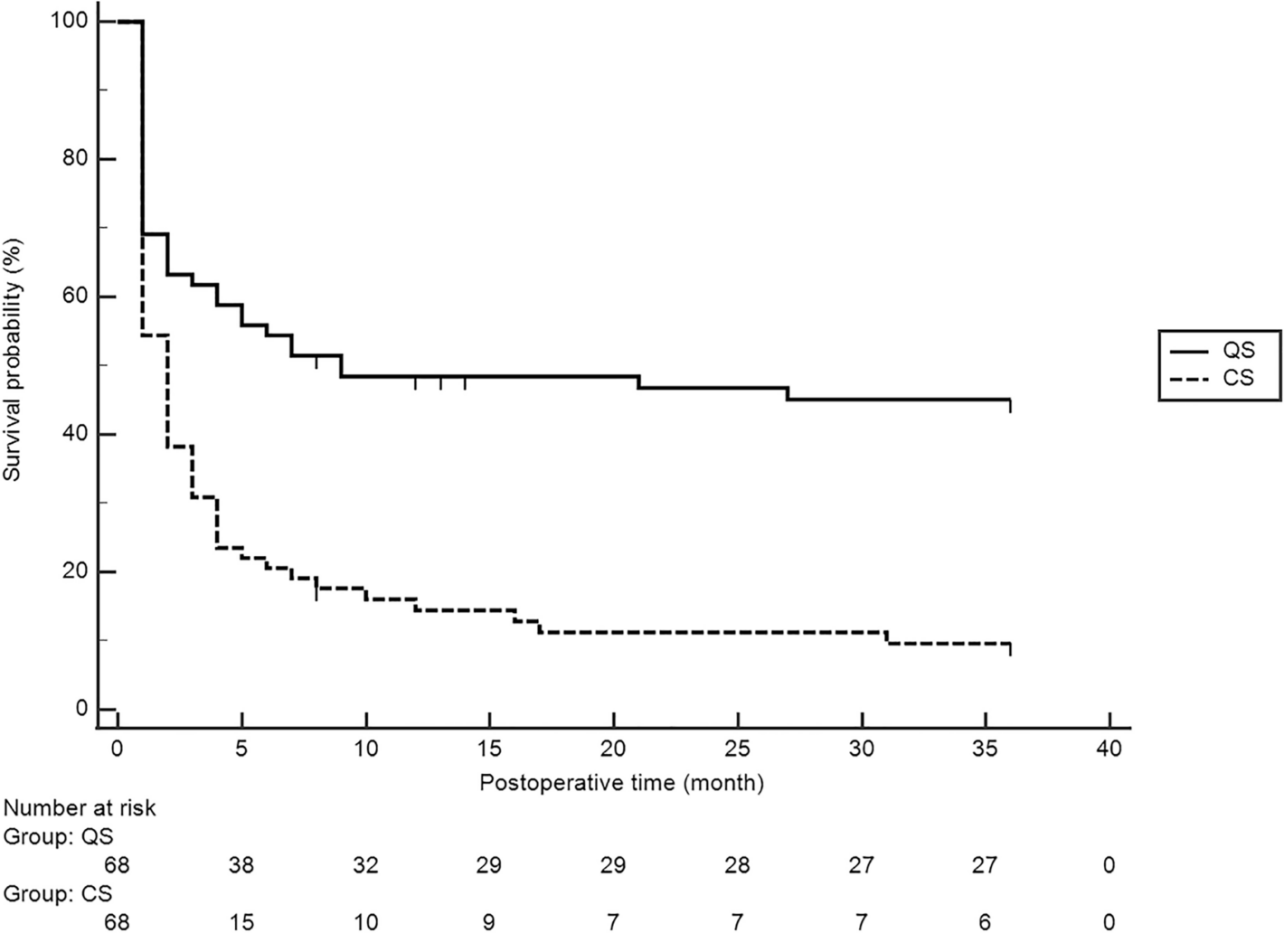
Kaplan–Meier survival curves for the three-year success rate of surgical bleb revision (SBR). The solid black line represents qualified success (QS), and the dotted line represents complete success (CS). The QS rates at 1, 2, and 3 years after surgery were 48.4%, 46.8%, and 45.1%, respectively. The CS rates at 1, 2, and 3 years after surgery were 14.4%, 11.2% and 9.6%, respectively

**Fig. 3:**
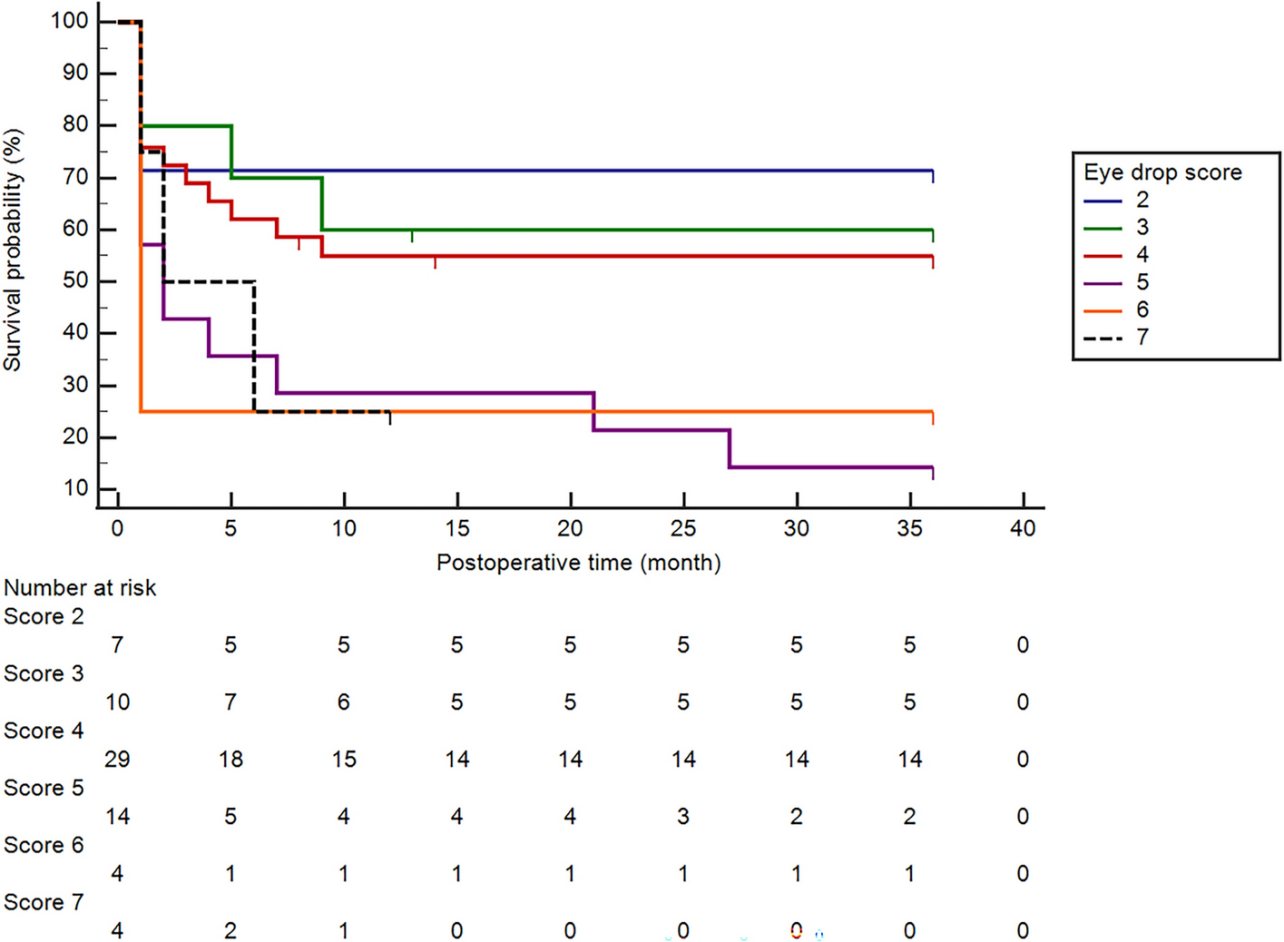
Kaplan–Meier survival curves for QS rate by the numbers of glaucoma medications used before initial trabeculectomy. The blue line shows the survival curve for patients with two glaucoma medications (n = 7), the green line for those with three (n = 10), the red line for those with four (n = 29), the purple line for those with five (n = 14), the orange line for those with six (n = 4), and the dotted black line for those with seven (4). The 3-year QS rates were 71.4%, 60.0%, 55.5%, 13.4%, and 25.0% for the eye with 2, 3, 4, 5, and 6 glaucoma medications used before TLE. No eye with seven glaucoma medications survived up to 3 years after SBR

Figure 2 shows Kaplan–Meier survival analysis. The success rates for QS at 1, 2, and 3 years after surgery were 48.4, 46.8, and 45.1%, respectively. The success rates for CS at 1, 2, and 3 years after surgery were 14.4, 11.2 and 9.6%, respectively. Cox proportional-hazards regression using stepwise method including age, pre-TLE IOP, pre-SBR IOP, interval between TLE and SBR, number of glaucoma medication before TLE, and baseline MD as covariates revealed that only the number of glaucoma medication used before TLE contributed to the survival prediction. An increase of one glaucoma medication increased the hazard ratio for surgical failure by 1.43-fold (95% confidence interval 1.07 to 1.92, *P* = 0.018). Figure 3 shows Kaplan Meier survival curves by the number of glaucoma medications before TLE. The 3-year QS rate for the patients who used 2, 3, 4, and 5 glaucoma medications before TLE was 71.4%, 60.0%, 55.0%, and 14.3%, respectively. There were 8 patients with 6 or more glaucoma medications prior to TLE, seven of them failed within one year after SBR, and only one survived at 3 years after SBR. The 3-year QS rate for the patients who used 4 or less glaucoma medications before TLE (n = 46) was 58.5%, while that for the patients with 5 or more glaucoma medications before TLE (n = 22) was 16.4% (*P* = 0.001, Logrank test).

Thirty-seven of the 68 included eyes failed within 3 years of surgery; 22 of the 37 failed eyes underwent additional glaucoma surgery, including 9 eyes with repeat TLE, 7 eyes with Ahmed glaucoma valve, 4 eyes with Baerveldt glaucoma implant, one eye with laser cyclophotocoagulation, and 1 eye with laser cyclophotocoagulation followed by Ahmed glaucoma valve. Data from 15 patients who failed within 3 years but did not undergo additional surgery are presented in Supplementary Table 1. In most cases, IOP eventually decreased and settled with eye drops and massage, and surgery was not required. There were five cases of progressive visual field loss, but in three cases the patients did not want additional surgery or did not return. In another case, surgery was not performed because treatment of the contralateral eye took precedence. In the other case, additional surgery was not performed because the eye had developed bullous keratopathy. Excluding these five cases, 41 patients (60.3%) were spared from additional glaucoma surgery for 3 years after SBR.

**Fig. 4:**
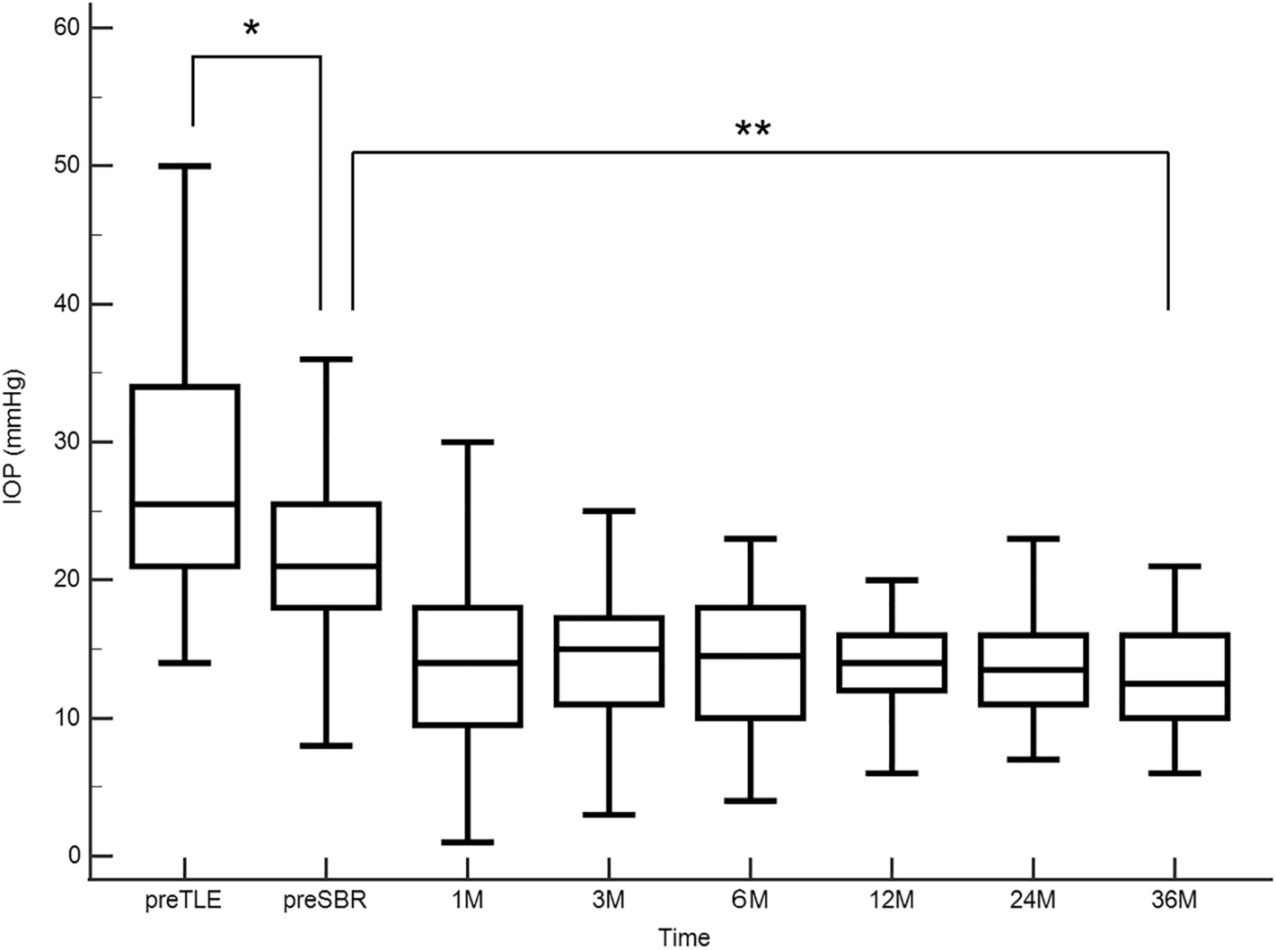
Box-and-whisker plots showing changes in IOP over time. Pre-SBR IOP was significantly lower than pre-TLE IOP (*P* = 0.0001, Wilcoxon test), and IOP decreased significantly after SBR (*P* < 0.0001, mixed effect model). *IOP* intraocular pressure, *SBR* surgical bleb revision, *TLE* trabeculectomy

**Fig. 5:**
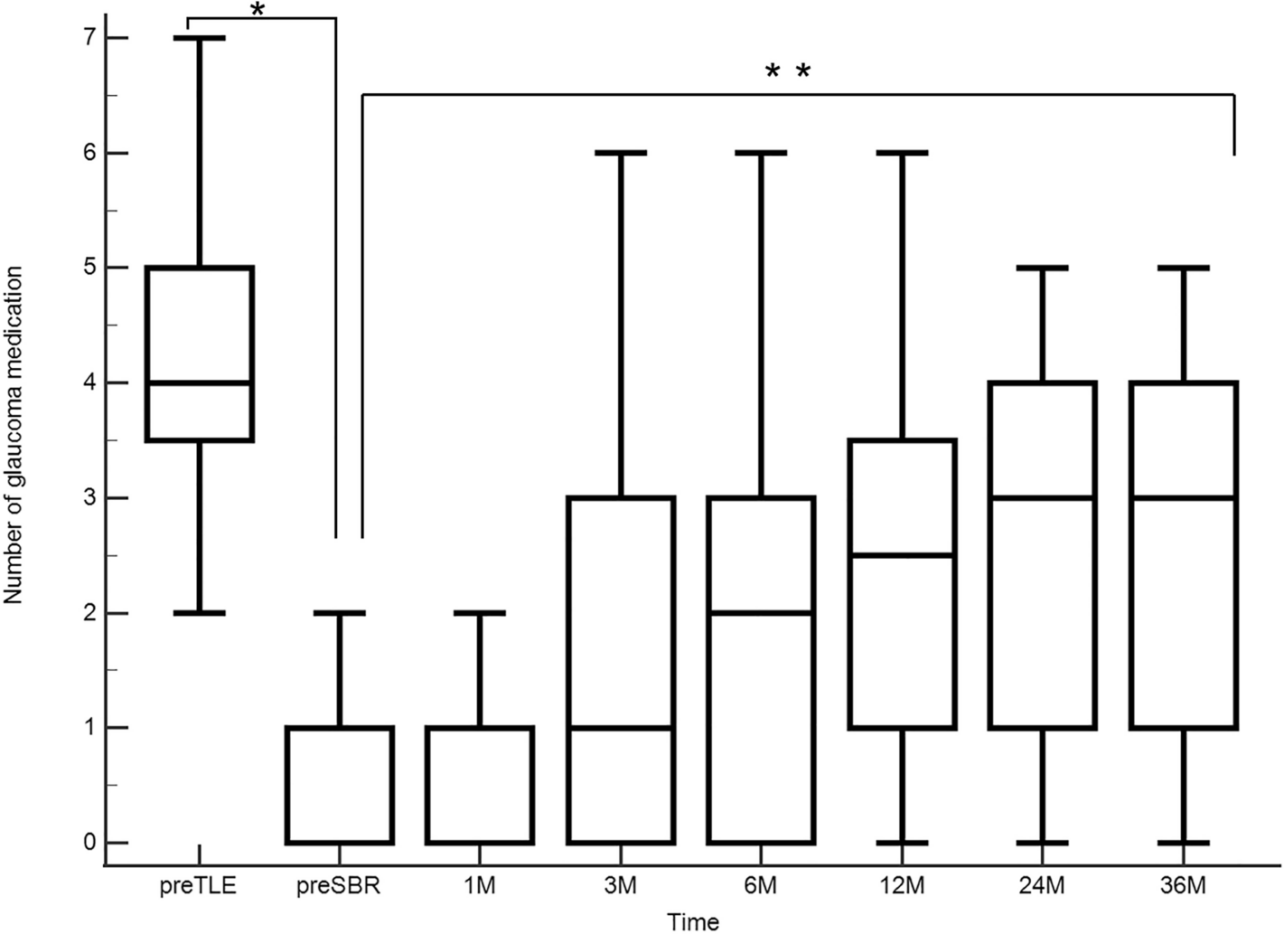
Box-and-whisker plots showing changes in the number of glaucoma medication used overtime. The number of medications used before SBR was significantly lower than that before TLE (*P* < 0.0001, Wilcoxon test). Glaucoma medications were reintroduced early postoperatively after SBR, and their number increased with postoperative time (*P* < 0.001, Mixed effect model)

Figures 4 and 5 show changes over time in IOP and the number of glaucoma medications used. Pre SBR IOP was significantly lower than IOP before TLE (*P* < 0.001, Wilcoxon test), and IOP decreased significantly after SBR (*P* < 0.0001, Mixed effect model). At the time of SBR, most patients were using post-TLE eye drops (steroids and antibiotics) and a few were using glaucoma medications. However, glaucoma eye drops were reintroduced early postoperatively after SBR, and the number increased with postoperative time (*P* < 0.001, Mixed effect model).

Table 2 compares pre- and postoperative IOP, number of glaucoma medications, MD, BCVA, and ECD. Thirty-one eyes that achieved QS at 3 years after SBR were analyzed. The median (IQR) IOP before SBR in all included patients was 21.0 (18.0–25.5) mmHg and was significantly lower than the pre-TLE value, which was 25.5 (21.0–34.0) mmHg (Wilcoxon test, *P* < 0.0001). However, since in this study SBR is a series of treatments with TLE, the pre-TLE IOP was used as the baseline. Similarly, pre-TLE data were used as the baseline for the number of glaucoma medication, MD, BCVA, and ECD. Because of the short interval between TLE and SBR, some patients had no data on MD, BCVA, and ECD values at the time of SBR. As a result, IOP, the number of glaucoma medications, and ECD significantly decreased from the baseline at 3-year post-SBR. BCVA also decreased postoperatively, but MD did not change from the baseline. Note that both preoperative and postoperative data for MD were available for only 15 eyes.

**Table 2:** Comparison before and after surgery

	Pre TLE	Three years post SBR	*P* value*
IOP (mmHg)	24.0 (20.0–31.0)	11.0 (10.0–16.0)	< 0.01
Number of glaucoma medications	4.0 (3.0–4.0)	2.0 (1.0–3.0)	< 0.01
HFA30-2 MD (dB)	– 19.97 (– 26.97 to – 15.61)	– 19.55 (– 23.13 to – 12.53)	0.6
BCVA	0.00 (– 0.08 to 0.26)	0.00 (0.00–0.46)	< 0.05
ECD (cell/mm^2^)	2545 (1903–2692)	2283 (1568–2526)	< 0.01

Data are expressed as median (interquartile range)

*TLE* trabeculectomy, *SBR* surgical bleb revision, *IOP* intraocular pressure, *HFA* Humphrey visual field, *MD* mean deviation, *BCVA* best corrected visual acuity, *ECD* corneal endothelial cell density.

*Wilcoxon signed-rank test

There were no serious complications during or after SBR. There was no loss of light perception. Complications seen were, transient hypotony with choroidal detachment in 14 eyes, temporary decrease in visual acuity because of hemorrhage spread to the vitreous cavity in 2 eyes, and vitreous opacity due to underlying uveitis in one eye. There were 15 eyes with bleb leak after SBR, but all recovered soon after with either eye ointment or additional suture or both. Shallowing of anterior chamber was seen in 2 eyes but resolved within a month. One eye with uveitic glaucoma developed bullous keratopathy 13 months after SBR (Supplemental Table 1). In this eye, corneal endothelial cells were already reduced before TLE.

Age, type of glaucoma (proportion of POAG), intervals between TLE and SBR, IOP before TLE and SBR, number of glaucoma medications used before TLE, pre-TLE BCVA, and pre-TLE MD were compared between the QS group (n = 31) and the failed group (n = 37) (Table 3). The number of glaucoma medications used before TLE was significantly larger in the failed group than in the QS group (*P *< 0.01, Mann Whitney U test). There was no significant difference between the two groups in other parameters.

**Table 3:** Comparison of failed and QS group

	Failed (n = 37)	QS (n = 31)	*P* value
Age (years)	69.0 (60.0–76.0)	73.0 (63.5–77.5)	0.36^†^
Types of glaucoma (n (%) POAG)	14.0 (20.6)	16.0 (23.5)	0.33^‡^
Intervals from TLE to SBR (months)	2.0 (1.0–3.0)	2.0 (2.0–3.0)	0.21^†^
Pre SBR IOP (mmHg)	22.0 (18.0–26.0)	21.0 (18.0–24.5)	0.81^†^
Pre TLE IOP (mmHg)	26.0 (21.0–36.0)	24.0 (20.0–31.0)	0.25^†^
Pre TLE Glaucoma Medications (n)	4.5 (4.0–5.0)	4.0 (3.0–4.0)	< 0.01^†^
Pre TLE BCVA (logMAR)	0.15 (0.00–0.30)	0.00 (– 0.08 to 0.26)	0.17^†^
Pre TLE HFA 30-2 MD (dB)	– 18.53 (– 26.78 to – 13.99)	– 19.58 (– 26.85 to – 12.86)	1.00^†^

Data are expressed as median (interquartile range)

*QS* qualified success, *POAG* primary open angle glaucoma, *TLE* trabeculectomy, *SBR* surgical bleb revision, *IOP* intraocular pressure, *BCVA* best corrected visual acuity, *logMAR* logarithm of the minimum angle of the resolution, *HFA* Humphrey visual field, *MD* mean deviation

^†^Mann Whitney Test

^‡^Fisher's exact test

## Discussion

At Kobe University, early interventions for inadequate filtration after TLE include topical steroids, laser suture lysis and ocular massage to increase filtration of aqueous humor. Even with such interventions, there are cases of early scarring of the bleb resulting in re-elevation of IOP, and for such cases we perform SBR within a few months post-TLE. To our knowledge, this is the first report on SBR performed for early scarring blebs after TLE. In this study, the three-year QS rate of SBR was 45.1%, with only 9.6% achieving CS, and many patients needed to resume glaucoma medications early postoperatively. Previous reports of SBR for uncontrolled IOP after TLE reported success rates ranging from 44 to 91% [20–24] although surgical techniques and definitions of success vary. In these reports, SBR was performed several years after TLE, whereas in our study, SBR was performed at a median of 2 months after TLE. Therefore, the patient population in the previous reports may differ from our study population, in which bleb scarring developed very early after TLE. Kokubun et al. report that in patients with POAG and neovascular glaucoma, early postoperative morphological changes in the bleb that may suggest scarring of the bleb were closely associated with survival of the filtering bleb at 1 year after TLE [2, 3]. The patients in our study developed bleb scarring very early after TLE and may have been a poor prognostic group for TLE in the first place, which may explain why they required early resumption of eye drops to lower IOP even after SBR. Patients whose IOP fell outside the criteria on two consecutive postoperative visits were considered failures, but some of the failed patients’ IOP eventually settled with medication and massage, and additional surgery was not performed in most cases. Forty-one patients (60.3%), excluding 5 patients who did not undergo recommended additional surgery for various reasons, were spared from additional surgery for at least 3 years after SBR. SBR may be an effective procedure given that 60% of cases of early bleb scarring that would otherwise have resulted in unsuccessful TLE could be maintained without additional surgery for the next 3 years.

Patients who had been using many glaucoma medications prior to TLE had poorer SBR results. Multiple or prolonged glaucoma medication use is reported to be a risk factor for TLE failure [26–28]. Preservatives in glaucoma medications are reported to increase conjunctival fibroblasts, macrophages, and lymphocytes and decrease goblet cells. These conjunctival changes caused by glaucoma medications may contribute to the early scarring of blebs, which, in addition to deteriorated outflow function, contribute to surgical failure. The median number of glaucoma medications used before TLE for the subjects in this study was four, making them a poor prognostic group for TLE in the first place. Among them, those with a further number of drugs exceeding 5 were found to have a low success rate in SBR. Therefore, in cases where the number of medications exceeds 5, SBR is likely to be ineffective, and additional surgery may be considered.

Bleb needling or revision using bleb knife may be more commonly performed for early bleb scarring after TLE than our SBR. The results of this study do not allow a direct comparison between bleb needling and SBR. Compared to needling, the advantages of SBR are that there is no blind manipulation, the flap and the scarred tissue around the flap can be seen directly, and hemostasis is assured. Furthermore, when compared with the needling on the slit lamp, the procedure can be performed safely in the operating room on patients who cannot remain at rest or who are in poor general condition. On the other hand, the disadvantages of SBR are the patient's burden of returning to the operating room again early after TLE, that SBR is more invasive to the conjunctiva than needling, and the fact that it cannot be repeated easily like needling. Furthermore, it takes several weeks to a month from the time the scarring of the bleb is identified, and the patient is scheduled for surgery to the time the SBR is performed in the operating room, during which time the scarring further develops. At Kobe University Hospital, when TLE is performed, it is explained in advance that, if scarring develops one to several months after TLE SBR may be performed in the operating room. Therefore, patients accept SBR. To minimize invasion of the conjunctiva, the conjunctival incision is made as small as possible posterior to the original flap. However, conjunctival incision and sutures may further exacerbate scarring. In this study many blebs re-scarred after SBR and the IOP rose again. In such cases, SBR was not repeated, glaucoma medications were resumed immediately, and in some cases, ocular massage was performed to lower IOP. With these approaches, some patients were able to achieve their target IOP without additional surgery for 3 years after SBR, even though their IOP exceeded the criteria for some time after SBR (Supplemental Table 1).

There were no serious complications from SBR. Although there was anterior chamber shallowing, bleb leak, hypotony and choroidal detachment after SBR, all these improved in a short period of time. In QS patients, VF remain unchanged at 3 years after SBR, but BCVA and ECD decreased. However, the short duration of TLE and SBR suggests that both are responsible for the decrease of vision and ECD. Overall, SBR was considered a safe procedure.

Limitations of this study are its retrospective nature, small sample size, and the fact that indications for SBR and additional surgery may differ between surgeons. Evaluation of bleb scarring after TLE was based on the findings of each surgeon; specific evaluation criteria were not used, nor were detailed evaluations of bleb using OCT or UBM, which may have led to different judgments by different surgeons. Further research is needed on the treatment efficacy of SBR.

In conclusion, SBR may be an effective treatment option for some cases of early scarring after TLE, but the surgical outcome is poor in patients with a high number of preoperative glaucoma medications.

## Supplementary Information

Below is the link to the electronic supplementary material.

Supplementary file1 (XLSX 12 KB)
